# Genetic analysis of novel phenotypes for farm animal resilience to weather variability

**DOI:** 10.1186/s12863-019-0787-z

**Published:** 2019-11-12

**Authors:** Enrique Sánchez-Molano, Vanessa V. Kapsona, Joanna J. Ilska, Suzanne Desire, Joanne Conington, Sebastian Mucha, Georgios Banos

**Affiliations:** 1 The Roslin Institute and R (D) SVS, University of Edinburgh, Easter Bush, Edinburgh, EH25 9RG UK; 2 Scotland’s Rural College, The Roslin Institute Building, Easter Bush, Edinburgh, EH25 9RG UK; 3 Poznan University of Life Sciences, 33 Wolynska, 60-637 Poznan, Poland

**Keywords:** Animal resilience, Climate change, Selective breeding, Heritability, Candidate genes

## Abstract

**Background:**

Climate change is expected to have a negative impact on food availability. While most efforts have been directed to reducing greenhouse gas emissions, complementary strategies are necessary to control the detrimental effects of climate change on farm animal performance. The objective of this study was to develop novel animal resilience phenotypes using reaction norm slopes, and examine their genetic and genomic parameters. A closely monitored dairy goat population was used for this purpose.

**Results:**

Individual animals differed in their response to changing atmospheric temperature and a temperature-humidity index. Significant genetic variance and heritability estimates were derived for these animal resilience phenotypes. Furthermore, some resilience traits had a significant unfavourable genetic correlation with animal performance. Genome-wide association analyses identified several candidate genes related to animal resilience to environment change.

**Conclusions:**

Heritable variation exists among dairy goats in their production response to fluctuating weather variables. Results may inform future breeding programmes aimed to ensure efficient animal performance under changing climatic conditions.

## Background

According to the United Nations Intergovernmental Panel on Climate Change, human activities since the pre-industrial times have had a strong impact on climate. Agriculture is believed to contribute to climate change mostly due to greenhouse gas emissions through the use of fertilisers, methane production by livestock and nitrous oxide emissions from soils [1]. Furthermore, indirect consequences of the agricultural industrialisation such as deforestation [2], intensive monoculture leading to a reduction in variation [3], and the improper use of water irrigation and industrial machinery have also contributed to climate change. The substantial rise in global atmospheric temperature has been particularly steep over the past few decades (0.17 °C/decade), and has been largely noticeable in the northern hemisphere during spring and winter and more uniform throughout the year in the southern hemisphere. In Europe, in addition to the gradual increase in temperature, climate change has also been manifesting in alterations in intra-seasonal and inter-annual variability, with decreasing variability of winter mean temperatures and increased variability of summer mean temperatures [4]. An increase in temperature variability is also predicted for tropical countries [5]. Additional modifications in precipitation and humidity patterns are also evident, with increased and decreased annual precipitation in northern and southern Europe, respectively, and fluctuations in precipitation in central Europe [6].

With regards to agriculture and livestock farming, the main focus to-date has been on mitigating the effects of methane and other greenhouse gas emissions [7, 8]. At the same time, there is a growing concern that climate change may adversely affect the quality and quantity of both plant [9] and livestock [10] products leading to reduced food availability as well as increased frequency and severity of disease [11]. Therefore, there is a recognised need to address the current detrimental effects of environmental degradation on animal and plant production, and to develop additional strategies to mitigate the problem [10, 12, 13].

Selective breeding for enhanced animal resilience to environmental variation may offer a novel strategy to address the impact of climate change on livestock production [14, 15]. The few genetic studies conducted to-date have focussed on extreme directional climate challenges such as heat stress from very high temperatures [16–19]. While these considerations are appropriate in specific geomorphological areas, climate change leading to increased seasonal variability in weather conditions may also affect animal performance [10, 12], even within the moderate temperature range.

Animal resilience must be properly defined in order to derive appropriate phenotypes across the range of prevailing and expected environmental conditions [20–22]. These phenotypes could be included in selective breeding programmes aiming at sustainable animal production levels in presence of environmental (climate) perturbations.

Different theoretical frameworks have been used to model resilience to environmental changes and its effects on animal performance. Recent studies have shown the potential use of genotype by environment interaction (GxE) to estimate resilience phenotypes for animal production traits [23–25]. In this context, individual phenotypes can be described as a continuous function of an environmental variable using random regression model approaches [26]. Reaction norm functions can then be used to express resilience as a phenotypic response of animal performance to changing environment (for example, weather).

The objectives of the present study were to (i) derive novel animal resilience phenotypes based on milk production changes in response to weather variability and (ii) investigate the genetic and genomic architecture of these newly derived animal phenotypes.

We deployed reaction norm functions to derive resilience phenotypes, mixed models for statistical analyses to estimate relevant genetic parameters, and genome-wide association studies to detect molecular markers and candidate genes controlling resilience. We used data from a well-monitored dairy goat population but our approach is applicable to any livestock species.

## Results

### Animal performance records and weather measurements

Descriptive statistics of animal performance and weather records are presented in Table 1. Daily milk yield, temperature and a temperature-humidity index (THI) reflected averages of a 10-day period.

**Table 1:** Descriptive statistics of animal performance and weather records

	DMY (kg)	T (°C)	RH (%)	THI
Mean	3.58	9.89	83.05	50.48
SD	1.51	4.62	5.35	7.62
Minimum	0.20	−3.27	65.65	29.13
Quantile 25	2.50	6.04	79.38	44.41
Median	3.50	9.99	83.13	50.95
Quantile 75	4.60	14.14	86.73	57.52
Maximum	12.60	18.92	97.27	65.25

Daily milk yield (DMY), daily temperature (T), daily relative humidity (RH) and daily temperature-humidity index (THI)

The prevailing weather conditions in the geographic region during the time of the study are illustrated in Additional file 1: Figure S1. These conditions are concordant with other weather reports in the UK [27], with average temperatures of 17-20 °C in July–August and 3-4 °C in January–February.

### Individual animal resilience phenotypes

Descriptive statistics of animal resilience phenotypes are shown in Table 2. These phenotypes reflect the change of individual animal daily performance (milk yield) in response to changing weather (temperature and THI). Values of individual phenotypes were both positive, suggestive of increased milk production at higher values of the weather measurement, and negative, reflecting decreased animal performance at higher values of the weather measurement. An additional phenotype was the absolute value of these records indicating stable (values close to zero) versus volatile milk production response to weather change.

**Table 2:** Descriptive statistics of resilience phenotypes expressed as milk production change (kg) per unit increase in weather variables

Phenotype	Mean	SD	Min	Max	K	S
T	0.029	0.048	−0.170	0.372	1.31	0.23
abs (T)	0.044	0.035	2.28E-6	0.372	3.03	1.38
THI	0.018	0.029	−0.102	0.224	1.31	0.22
abs (THI)	0.026	0.021	5.44E-7	0.224	3.02	1.38

*T* Performance change per unit of temperature (°C) change, *THI* Performance change per unit of temperature-humidity index change, *abs* Absolute value of corresponding performance change, *SD* Standard deviation, *K* Kurtosis, *S* Skewness

### Genetic parameters of resilience phenotypes

Variance components and genetic parameter estimates for animal resilience phenotypes are shown in Table 3. All estimates were significantly greater than zero (*P* < 0.01). Genetic correlation of total lifetime milk yield with the resilience phenotypes related to absolute slopes (volatility phenotypes) were also significantly positive (*P* < 0.01). The latter implies an unfavourable correlation where animals with high milk yield potential are also more likely to have their milk production affected by change in changing weather.

**Table 3:** Genetic parameters of resilience phenotypes expressed as milk production change per unit increase in weather variables

Phenotype	V_P_	V_A_	h^2^	r_A_
T	2.06E-3 ± 2.27E-5^*^	2.18E-4 ± 2.79E-5^*^	0.11 ± 0.01^*^	0.05 ± 0.07
abs (T)	6.70E-3 ± 7.30E-5^*^	6.13E-4 ± 8.68E-5^*^	0.09 ± 0.01^*^	0.46 ± 0.07^*^
THI	7.51E-4 ± 8.27E-6^*^	7.92E-5 ± 1.01E-5^*^	0.11 ± 0.01^*^	0.04 ± 0.07
abs (THI)	4.03E-3 ± 4.39E-5^*^	3.63E-4 ± 5.20E-5^*^	0.09 ± 0.01^*^	0.46 ± 0.07^*^

*T* Performance change per unit of temperature (°C) change, *THI* Performance change per unit of temperature-humidity index change, *abs* Absolute value of corresponding performance change (square root transformed), *V*_*P*_ Phenotypic variance, *V*_*A*_ Additive variance, *h*^*2*^ Heritability, *r*_*A*_ Genetic correlation with total lifetime milk yield (square root transformed)

### Genomic association analysis

**Fig. 1:**
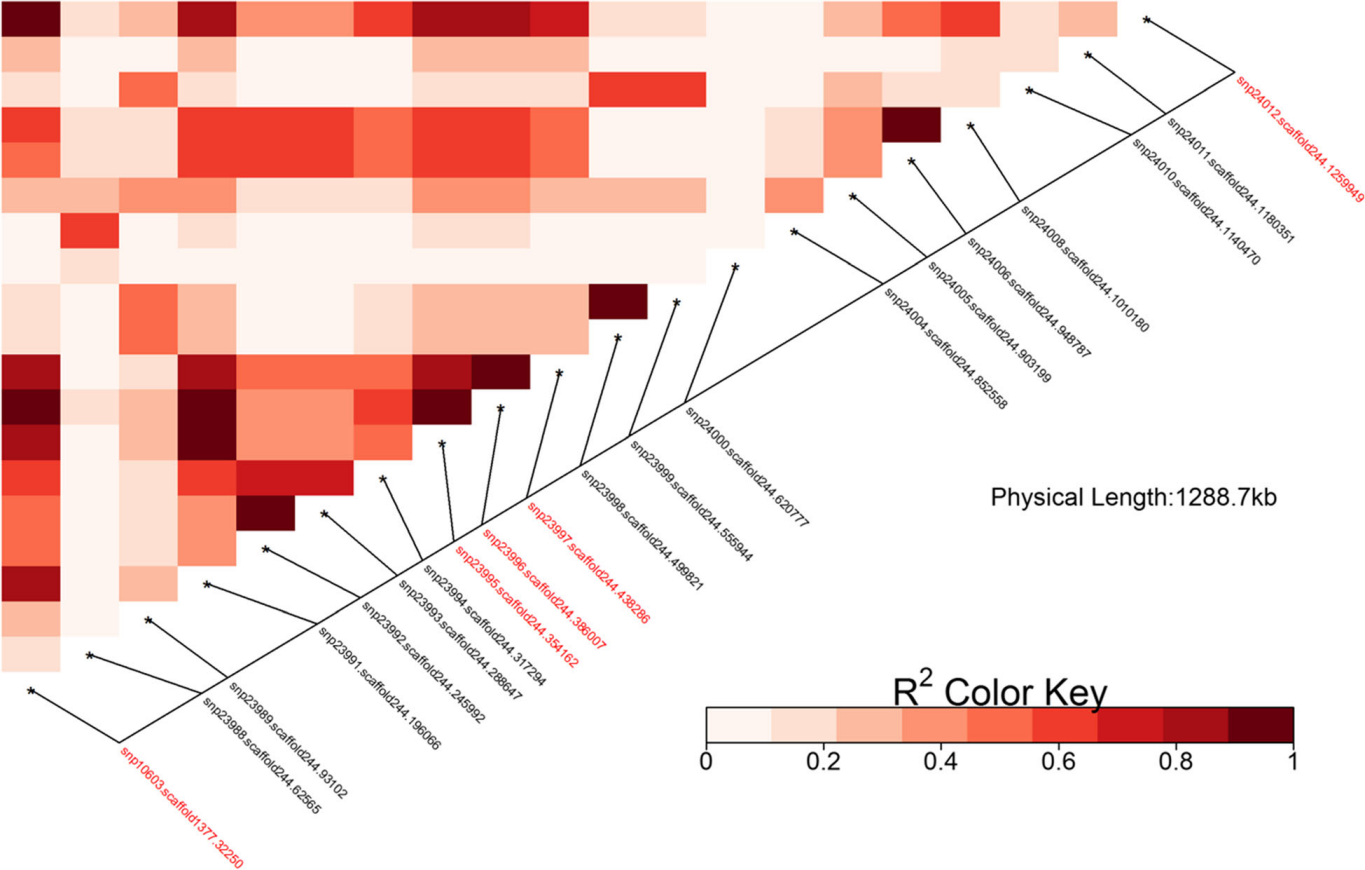
Linkage Disequilibrium structure on chromosome 19 spanning region between significant SNP for resilience phenotypes: significant SNP are marked in red

Population structure was not detected and values of the inflation factor *λ* ranged from 0.996 to 1.001 for all analysed phenotypes. Several single nucleotide polymorphisms (SNPs) were found to be significantly associated with total lifetime milk yield and resilience phenotypes either at genome- or chromosome-wide levels (Table 4, Additional file 2: Figure S2). Table 4 summarises these results and includes annotated genes found in the respective genomic regions. One genome-wide significant SNP was detected on chromosome 19 for total lifetime milk yield and resilience phenotypes based on absolute slopes, which was also significant at chromosome-wide level for all the other resilience phenotypes. Another two genome-wide significant SNPs were detected for total lifetime milk yield on chromosomes 8 and 13, with no effect on resilience traits. Other chromosome-wide associations were detected on chromosomes 3, 4, 13, 14, 19, 20 and 24. All significant SNPs on chromosome 19 span a region of 1289 kb, representing a relatively high linkage disequilibrium block (Fig. 1).

**Table 4:** Single Nucleotide Polymorphisms significantly associated with goat milk yield and resilience phenotypes at genome-* and chromosome-wide level

Chr	Position (bp)	Trait	Beta effect ± SE	-log_10_(P)	pve (%)	Nearest genes
3	2,389,800	TMY	0.6580 ± 0.1501	4.93	0.18	HDAC4
4	42,384,455	TMY	0.7455 ± 0.1646	5.22	0.19	HECW1
8	39,875,253	TMY	1.1943 ± 0.1863	9.82*	0.37	SLC1A1; GLIS3
13	58,370,939	TMY	0.7424 ± 0.1484	6.24*	0.24	BMP7; TFAP2C
13	66,542,955	T	−0.0041 ± 0.0010	4.59	0.17	RPRD1B; LOC108637373
		THI	−0.0025 ± 0.0006	4.53	0.16	
14	69,473,160	TMY	−0.7232 ± 0.1597	5.22	0.19	LOC102186225
19	26,192,068	T	0.0040 ± 0.0009	4.64	0.17	TRNAS-UGA; KIF1C
19	26,578,715	T	0.0040 ± 0.0010	4.52	0.16	LOC102178853; ALOX12
19	26,610,550	T	0.0042 ± 0.0010	4.90	0.18	RNASEK
		abs (T)	0.0156 ± 0.0015	24.55*	0.91	
		THI	0.0024 ± 0.0006	4.60	0.17	
		abs (THI)	0.0120 ± 0.0012	24.12*	0.89	
		TMY	3.6668 ± 0.1916	79.53*	2.78	
19	26,662,221	T	0.0039 ± 0.0010	4.50	0.16	ASGR2
19	27,480,733	T	0.0043 ± 0.0009	5.40	0.20	ALOXE3
		THI	0.0025 ± 0.0006	5.13	0.19	
20	3,621,302	TMY	−0.7984 ± 0.1828	4.90	0.18	FGF18; SMIM23
20	10,173,528	TMY	0.8545 ± 0.1882	5.25	0.19	NAIP
24	52,818,318	abs (T)	−0.0074 ± 0.0017	4.74	0.17	LOC108633777; LOC108633778
		abs (THI)	−0.0058 ± 0.0013	4.66	0.17	

*TMY* Total lifetime milk yield, *T* Performance change per unit of temperature (°C) change, *THI* Performance change per unit of temperature-humidity index change, *abs* Absolute value of corresponding performance change, *Chr* Chromosome, *pve* Percentage of explained phenotypic variance. Genome-wide significances are indicated with an asterisk *

## Discussion

Climate change is expected to affect future livestock performance due to not only directional changes such as rising atmospheric temperature but also an increased volatility in weather conditions. Selective breeding for enhanced animal resilience to weather changes may contribute to the mitigation of the problem, leading to stable animal performance that is unaffected by weather variability. The present study set out to identify novel phenotypes of animal resilience and address their potential use in breeding schemes by estimating genetic parameters and identifying potential candidate genes. Results would determine how amenable animal resilience might be to improvement through genetic selection and how to inform relevant breeding programmes.

The use of linear slopes derived from reaction norm functions fitted to random regression models provided an assessment of the response of individual animal performance to changing weather including atmospheric temperature and THI. While the assumption of linearity was valid for the available range of weather measurements in the present study, other weather measurements and/or different value ranges of the same measurements in other geomorphological regions of the world might warrant investigation of non-linear models. In the latter case, the methodology presented here would still be relevant, as slopes at specific ranges in the weather measurement trajectory may be derived and used as distinct resilience phenotypes. For example, in areas where climate change is expected to lead to increased temperatures beyond the heat stress threshold (around 35°C for dairy goats [28]), slopes of performance traits below and above this threshold could be treated as separate phenotypes in a multi-trait breeding index.

When considering a range of temperatures below the heat stress threshold, as was the case in the present study, low temperatures are associated with lower average animal performance. Under cold stress (temperatures below 10 °C), animal feed intake is mostly directed towards maintaining their thermal balance requirements at the cost of producing less milk. Under higher temperatures, but still below the heat stress threshold, thermal balance requirements will be reduced, leading to better animal performance. Indeed, population curves from the reaction norm in the present study revealed a favourable impact of rising temperature and THI on performance manifested as increased daily milk production. The effect of THI almost mirrored that of temperature, partly because of the formula used to estimate THI [29] and partly because a relatively wider range of temperature values was observed in our data compared to humidity.

The individual animal resilience phenotypes derived in the present study exhibited significant phenotypic variation. Thus, the same weather change invoked positive or negative responses in different individuals while, for others, production was not affected at all (zero slopes). The latter individuals could be considered as the most resilient to climate change.

Furthermore, a significant proportion of the observed phenotypic variation among animals was genetic and heritable. Heritability estimates for resilience phenotypes ranged from 0.09 to 0.11, which is within the range of estimates for other fitness-related traits previously reported in goats [27], cattle [28] and sheep [29]. Although relatively low, these estimates are significantly greater than zero meaning that animal resilience to weather change is amenable to improvement via selective breeding. Since the outcome of selective breeding is cumulative, it is recommended that relevant programmes be put in place immediately in order to gradually and systematically enhance animal resilience to weather conditions as climate change becomes more pronounced.

When resilience was defined as the absolute value of the slope, reflecting volatility of animal performance with changing weather, a significant antagonistic genetic correlation with the actual level of milk production was estimated. This correlation suggests that animals with the genetic capacity for high milk yield will also be genetically predisposed to less stable milk production when challenged with changing weather. Although our range of temperatures is outside the heat stress interval, this result is also in agreement with previous studies on heat stress, where high merit animals were found to be more susceptible to environmental change [30, 31]. Therefore, careful consideration of resilience phenotypes should be made when including these traits in the breeding goal, in order to properly account for potentially unfavourable correlations with other traits of interest. Selection index theory can be used to effectively combine genetically antagonistic traits leading to overall genetic improvement in livestock [32–35]. Furthermore, index weights will need to be re-estimated every few generations in order to account for potentially new genetic correlations between traits emanating from changes in linkage disequilibrium due to selection.

Our genome-wide analyses identified several genomic markers associated with resilience phenotypes, particularly on chromosome 19. Although the significant SNPs identified on this chromosome were in mid to high linkage disequilibrium, only three of them, positioned within less than 0.1 Mb of each other, defined an actual haplotype with an overall squared correlation greater than 0.8 [36]. In this haplotype, one genome-wide significant SNP was detected affecting milk production level and relevant resilience phenotypes based on absolute slopes, which was also significant at chromosome-wide level for all the other resilience phenotypes. A previous study in goats [37] has shown genome-wide significant association of milk yield with another SNP in the same region located within 32 kb from the SNP identified here. Our SNP was found in exon 3 of the *RNASEK* gene, which encodes ribonuclease K protein. While the particular function of the latter is unknown, other ribonuclease pathways have been previously shown to be related to milk production [38] as well as host defence tissues and secretions in cattle [39]. Furthermore, ribonucleases are often involved in detention of protein synthesis to conserve energy under stress conditions [40]. Other chromosome-wide significant SNP associations for slopes on temperature and THI were also detected in this haplotype on chromosome 19, close to genes *ALOX12* and *ASGR2*. Gene *ALOX12* encodes the arachidonate 12-lipoxygenase, previously linked to goat milk and protein yield [37, 41], and the development and maintenance of the skin barrier [42]. Gene *ASGR2* encodes a subunit of the asialoglycoprotein receptor, associated with udder attachment in goats and cattle [37, 43].

Additional SNPs affecting resilience phenotypes were found on chromosome 19 outside the defined haplotype. Of particular interest is the association close to *ALOXE3*, which encodes the arachidonate lipoxygenase 3, a protein implicated in skin differentiation. In humans, mutations of this gene cause congenital ichthyosis, a skin disease with several symptoms including intolerance to heat and humidity [44]. Furthermore, this protein is also involved in the development of fat cells, and was previously linked to udder depth in goats [37] and to the pathway of arachidonic acid, a polyunsaturated fatty acid present in mammals’ milk [45].

Furthermore, several SNPs were detected significantly affecting total lifetime milk yield in the present study without any significant association with resilience. Among these SNPs, a genome-wide significant SNP was detected on chromosome 8, previously associated with goat milk production [37] and close to genes *SLC1A1* and *GLIS3*. Another genome-wide significant SNP was detected on chromosome 13, close to genes *BMP7* and *TFAP2C*, with the latter (transcription factor AP-2 gamma) having been previously associated to mammary development and several milk traits in sheep [46]. Other chromosome-wide significant SNPs for milk yield were detected on chromosomes 3, 4, 13, 14 and 20. The region on chromosome 4, in particular, is between exons 1 and 2 of gene *HECW1*, previously associated with vitamin B-12 content in cow milk [47].

Significant SNPs detected in the present study were generally located close to genes encoding proteins related to lipid metabolism, skin differentiation and stress response. Particular genetic variants segregating in the studied population could be related to variation in tolerance to temperature and humidity through a combination of direct effects on metabolism and indirect effects on stress and discomfort, even within the range of thermoneutral temperatures. However, identified SNPs explained only a small proportion of the trait variances, thus potentially indicating a polygenic architecture underlying resilience of animal milk production to weather change. Therefore, these results support the hypothesis of a complex underlying genetic link between animal production and environmental comfort involving multiple biological networks.

Additional considerations are warranted when addressing the impact of climate change on breeding schemes. Of particular importance are the strength and direction of the expected changes. In the case of the UK, temperatures are expected to rise by about 2 °C by 2100, with a potential 4.2 °C increase in summer temperatures in southern England by 2080 [48]. Under the same scenario, winters will become wetter by up to 23% by 2080 and summers drier by up to a 24%, with more frequent and severe droughts [48]. These changes will bring higher weather instability and impose a threat to animal performance if animal resilience is not considered in breeding schemes. Therefore, selective breeding schemes should include resilience phenotypes based on absolute values of slopes in order to select animals that are more resilient to short- and medium-term changes.

However, in other cases, the directionality of the individual animal production change might be more important than the effect of the increased variability. Resilience phenotypes based on actual slopes rather than their absolute values might then prove more useful, allowing to select animals that have an increased performance in the direction of the expected climate change. Example scenarios where these schemes would be useful are countries where the changes in weather will potentially lead to seasonal values in a particular direction. Selective breeding schemes could then be informed by multiple resilience phenotypes measured at different values of weather measurements (for example temperature), thus creating an animal index based on a combination of the directional increase in animal performance up to the inflection (stress) threshold and stability of performance thereafter.

Furthermore, an economic assessment of the reaction norm as a novel animal phenotype is important, particularly when combining multiple traits in selection indices. Previous studies [49, 50] have shown that the economic values of phenotypes derived with reaction norms depend on the trait whose stability in different conditions is measured as well as the diversity of environments where progeny of the selected animals will be raised. However, this consideration was out of the scope of this study, and further research needs to be conducted within the context of particular breeding schemes.

Finally, the use of reaction norms to derive resilience phenotypes can be applied not only to production traits, as shown in the present study, but also to other animal traits related with health and reproduction. While previous studies of fitness traits have not detected large genotype by environment interactions [51, 52] in dairy cattle, studies in beef cattle have revealed a significant impact of such interactions on animal reproduction [53]. Therefore, it is important to consider the possibility of a genetic basis of resilience in all biological functions of interest and the potential inclusion in selection indices for breeding schemes.

## Conclusions

The present study has demonstrated the applicability of reaction norms to obtain resilience phenotypes for animal performance to weather variability. Phenotypes obtained exhibited significant heritable genetic variance and can be used to underpin selective breeding schemes aiming to enhance animal performance and production stability in varying weather conditions. Candidate genes were detected for several resilience phenotypes, including genes related to stress response, lipid metabolism and skin development. These results can be used to further improve the accuracy of selective breeding. Non-linear models and a more extensive range of environments should be considered in future studies to account for variation outside the range studied here.

## Methods

### Data

Daily milk production records of individual animals were obtained from two UK dairy goat farms located at latitude 53° and 54° north. Strong genetic connectedness existed between the two farms as a result of inter-farm breeding program [54]. Animals in these farms are kept in an environment consisting of sheds without climate-controlled conditions. Because of specific management practices in these farms, daily animal milking records obtained were actually the average over 10 consecutive days.

Only records in the first 720 days of lactation were kept for the present study. Data were limited to goats that kidded from 2007 onwards, at a kidding age between 9 and 89 months and with at least three valid milk records. In addition, animal records with a lifetime estimate of the average daily milk yield outside three standard deviations from the mean were removed. The final dataset consisted of 980,689 milk records for 20,546 goats.

Animal pedigree was extracted from the farm database and comprised 46,825 animals spanning 19 generations, including 524 sires and 20,205 dams.

Weather data were obtained from the nearest weather station (less than 20 miles from the farms) and included average daily temperature and humidity. A temperature-humidity index (THI) was then calculated using the National Research Council formula [29]:

1 $$ \mathrm{T}\mathrm{HI}=\left(1.8\ast \mathrm{T}+32\right)-\left(0.55--0.0055\ast \mathrm{RH}\right)\ast \left(1.8\ast \mathrm{T}-26\right) $$

where THI = temperature-humidity index; T = average daily temperature (°C) and RH = average daily humidity (%). In consistence with the definition of animal performance, weather measurements used in the study represented averages of the same 10-day periods corresponding to each milk production record.

### Individual resilience phenotypes

A theoretical random regression model including a reaction norm function is:

2 $$ {y}_{ij}=X+f\left(\beta, {X}_j\right)+{f}_i\left({a}_i,{X}_j\right)+{e}_{ij} $$

where *y*_*ij*_ corresponds to the performance record of individual animal *i*, at a given environment *j*, *X* corresponds to a set of fixed effects describing all environments, *f*(*β*, *X*_*j*_) corresponds to a function (population reaction norm) describing the relationship between average animal performance and environment *j*, *f*_*i*_(*a*_*i*_, *X*_*j*_) corresponds to a function (individual animal reaction norm) describing the relationship between individual animal *i* and environment *j* (expressed as a deviation from the population reaction norm) and *e*_*ij*_ corresponds to the residual.

**Fig. 2:**
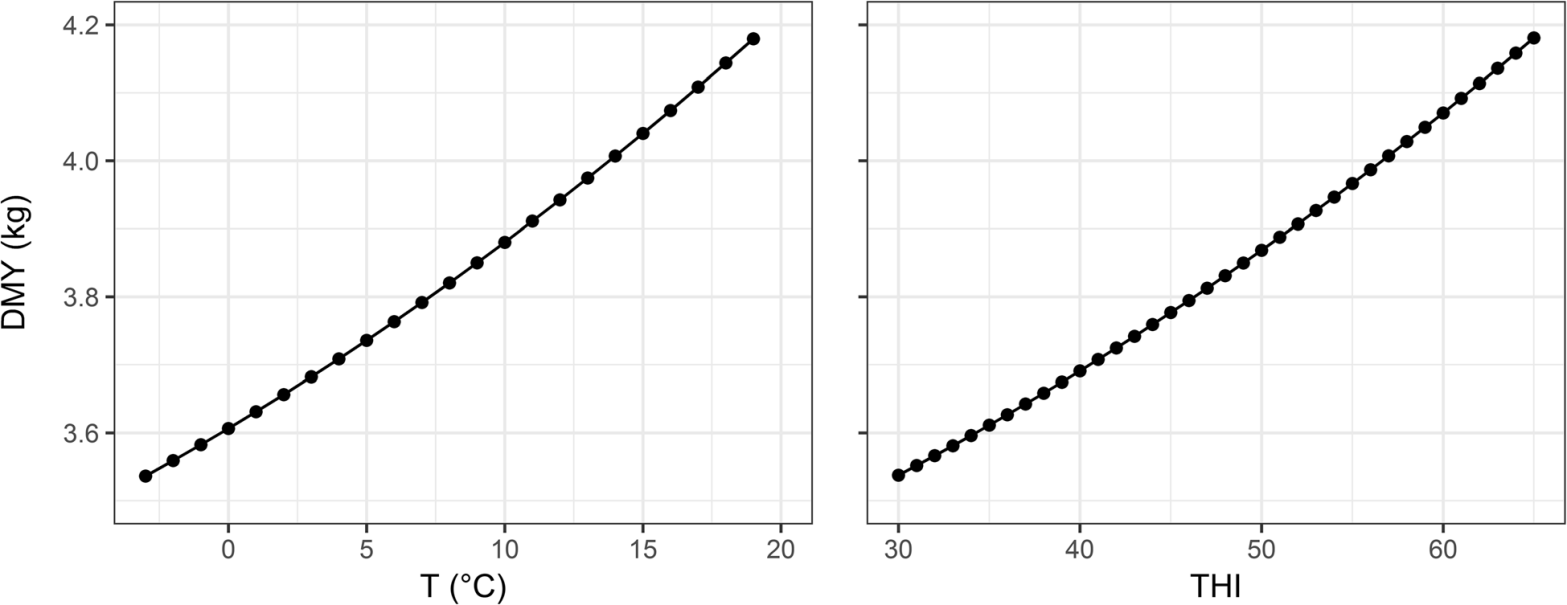
Population reaction norms: Daily milk yield evolution in response to temperature (T) and temperature-humidity index (THI) variability. Reaction norms were modelled with second degree Legendre polynomials

This model was fitted to milk yield records and corresponding temperature and THI values using second degree Legendre polynomials for the reaction norm function and the BLUPF90 suite of programs [55]. Initial exploration revealed a relatively linear behaviour for both weather measurements and animal performance (Fig. 2). Therefore, further analyses were conducted using the following simplified model with first degree Legendre polynomials [23]:

3 $$ {y}_{ij}=X+\mu +{\mu}_i+\left(s+{s}_i\right)\ast {X}_{ij}+{e}_{ij} $$

where *μ* corresponded to the population average intercept, *μ*_*i*_ corresponded to the animal *i* intercept deviated from the population intercept, and *s* and *s*_*i*_ corresponded to the population and individual *i* (as deviation) slopes on the fixed effect (environment); all other terms were as in model (2). The population reaction norm then was *μ* + *sX*_*ij*_ and the individual reaction norm *μ*_*i*_ + *s*_*i*_*X*_*ij*_, expressed as deviations from the population reaction norm.

Pedigree information was not included in model (3). Fixed effects included in this model were farm, interaction of calendar year and season of kidding, age at most recent kidding prior to milking date, number of days in milk, interaction between farm and date of record, and lactation (milking period) number.

Subsequently, individual reaction norms were computed per animal by adding the overall population norm to the corresponding individual animal deviation. Slopes of these individual reaction norms were estimated using derivatives, indicating the change in animal performance (milk yield) in response to weather fluctuations. These slopes were considered as the animal resilience phenotypes. Furthermore, absolute values of the estimated individual slopes were considered as additional resilience phenotypes, reflecting the stability/volatility of animal performance in relation to weather change, with values closer to zero representing more stable (resilient) animals.

### Genetic parameters of resilience phenotypes

Variance components and heritability estimates of all animal resilience phenotypes were derived from mixed models including the available pedigree information, using the ASReml software [56]. The distribution of resilience phenotypes based on the absolute value of slopes was normalised by applying a square root transformation. Fixed effects in the mixed models included total number of milking days, farm, total number of lactations, age at first kidding (onset of productive life) and interaction between calendar year and season of first kidding.

Univariate analyses were conducted for each resilience phenotype separately to estimate its additive genetic variance and heritability. Bivariate analyses of resilience with total milk produced throughout the animal’s productive life (square root transformed to normalise) were also conducted to estimate genetic correlations.

### Genomic association analysis of resilience phenotypes

A total of 10,620 animals with resilience phenotypes had been genotyped with the Illumina Caprine 50 K BeadChip containing 53,347 Single Nucleotide Polymorphisms (SNPs). Marker quality assurance removed SNPs on the sex chromosomes and those autosomal SNPs with Illumina GC score < 0.6, call rate < 95%, minor allele frequency < 0.05 and deviations from Hardy-Weinberg equilibrium (Bonferroni corrected threshold of 10^− 7^). Sample quality was assessed, and samples with call rates >90% were kept. These quality assurance edits resulted in a final set of 10,620 animals and 44,280 SNPs across all 29 autosomes with positions based on the most recent goat genome assembly ARS1 [57].

Association analyses were performed using the multi-locus mixed model algorithm [58] implemented in Golden Helix SNP & Variation Suite v8.8.3. The following model was used:

4 $$ \mathbf{y}=\mathbf{X}\beta +\mathbf{Za}+\mathbf{e} $$

where **y** was the vector of animal phenotypes for the analysed trait; ***β*** was a vector of coefficients for the SNP effects and other fixed effects (same as described for the estimation of genetic parameters); **a** was the vector of random animal polygenic effects; **e** was the vector of random residual effects; and **X** and **Z** were incidence matrices relating observations to fixed and random animal effects, respectively.

The vector of random animal effects **a** and residual effects **e** in model (3) were assumed to follow normal distributions with **a** ~ N $\Big(0,\mathbf{G}{\sigma}_a^2$) and **e** ~ N $\Big(0,\mathbf{I}{\sigma}_e^2$), where **G** corresponds to the genomic relatedness matrix, **I** corresponds to the identity matrix and ${\sigma}_a^2$ and ${\sigma}_e^2$ correspond to the genetic and residual variances, respectively. Covariance between **a** and **e** was assumed to be zero.

The genomic relationship matrix **G** was calculated following VanRaden [59].

$$ \mathbf{G}=\frac{\mathbf{SS}\hbox{'}}{2{\sum}_{i=1}^{\mathrm{N}}{\mathrm{p}}_{\mathrm{i}}\left(1-{\mathrm{p}}_{\mathrm{i}}\right)} $$

where **S** is a centred incidence matrix of SNP genotypes, *N* is the number of SNP markers, and p_i_ is allele frequency of marker *i*.

Statistical significance of SNPs was assessed using Wald tests. Following a forward-backward stepwise regression [58], once the algorithm performed an initial scan testing each marker, additional genome scans were performed adjusting the model to account for the most significant SNPs on the initial scan. Significance thresholds were set at both genome- and chromosome-wide levels using Bonferroni corrections for multiple marker testing with a significance level of *P* < 0.05. This resulted in a genome-wide significance threshold of -log_10_(*P*) **=** 5.95. For significant markers, the proportion of explained phenotypic variance (pve) was estimated as:

$$ \mathrm{pve}=\frac{{\mathrm{mrss}}_{\mathrm{h}0}-{\mathrm{mrss}}_{\mathrm{k}}}{{\mathrm{mrss}}_{\mathrm{h}0}} $$

where *mrss*_*h0*_ is the Mahalanobis root sum of squares for the null hypothesis and *mrss*_*k*_ is the Mahalanobis root sum of squares for marker *k*.

## Supplementary information

**Additional file 1: Figure S1.** Monthly average of weather measurements: daily temperature (T) and temperature-humidity index (THI). Standard deviations are shown as bars.

**Additional file 2: Figure S2.** Manhattan plots and QQ-plots: Total milk yield (A), performance change due to daily temperature change (B), performance change due to temperature-humidity index change (C), absolute value of performance change due to daily temperature change (D), absolute value of performance change due to THI change (E).

## Data Availability

The animal genotypes are commercially sensitive. For more information, please contact GB.

## Abbreviations

SNP: Single Nucleotide Polymorphism
THI: Temperature-Humidity Index
UK: United Kingdom
