# Supplementary figures and images for: Genetic analysis of novel phenotypes for farm animal resilience to weather variability
Source: BMC Genet. 2019 Nov 12;20:84. doi: 10.1186/s12863-019-0787-z (PMC6849266; doi:10.1186/s12863-019-0787-z)

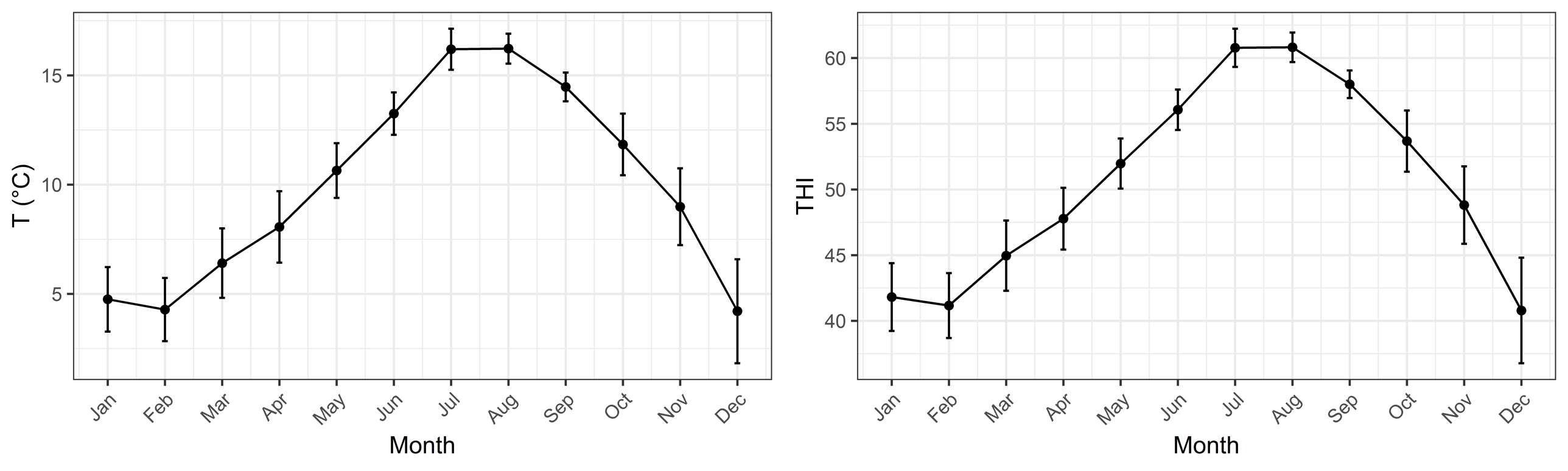

Supplement: Supplementary file 1 — Additional file 1: Figure S1. Monthly average of weather measurements: daily temperature (T) and temperature-humidity index (THI). Standard deviations are shown as bars. [file 12863_2019_787_MOESM1_ESM.tif]
